# Evaluation of MVCT images with skin collimation for electron beam treatment planning

**DOI:** 10.1120/jacmp.v9i3.2773

**Published:** 2008-06-23

**Authors:** Allen B. Beardmore, Isaac I. Rosen, Dennis A. Cheek, Robert S. Fields, Kenneth R. Hogstrom

**Affiliations:** ^1^ Department of Physics and Astronomy Louisiana State University and Agriculture & Mechanical College, Baton Rouge Baton Rouge LA USA; ^2^ LA and Mary Bird Perkins Cancer Center Baton Rouge LA USA

**Keywords:** electron therapy, skin collimation, megavoltage CT

## Abstract

This study assessed the potential of using megavoltage CT (MVCT) images taken with high density skin collimation in place for electron beam treatment planning. MVCT images were taken using the TomoTherapy Hi‐Art system (TomoTherapy Inc., Madison, WI), and the CT numbers were converted to density by calibrating the Hi‐Art system using an electron density phantom. Doses were computed using MVCT images and kVCT images and compared by calculating dose differences in the uniform dose region (>90%, excluding buildup region) and calculating distance‐to‐agreement (DTA) in high dose‐gradient regions (penumbra and distal falloff, 90%–10%). For 9 and 16 MeV electron beams of 10×10 cm calculated on a homogeneous CIRS Plastic Water (Computerized Imaging Research Systems Inc., Norfolk, VA) phantom without skin collimation, the maximum dose differences were 2.3% and the maximum DTAs were 2.0 mm for both beams. The same phantom was then MVCT scanned nine times with square skin collimators of Cerrobend on its surface ‐ field sizes of 3×3, 6×6, and 10×10 cm and thicknesses of 6, 8, and 10 mm. Using the Philips Pinnacle^3^ treatment planning system (Philips Medical Systems, N.A., Bothwell, WA), a treatment plan was created for combinations of electron energies of 6, 9, 12, and 16 MeV and each field size. The same treatment plans were calculated using kVCT images of the phantom with regions‐of‐interest (ROI) manually drawn to duplicate the sizes, shapes, and density of the skin collimators. With few exceptions, the maximum dose differences exceeded ±5% and the DTAs exceeded 2 mm. We determined that the dose differences were due to small distortions in the MVCT images created by the high density material and manifested as errors in the phantom CT numbers and in the shape of the skin collimator edges. These results suggest that MVCT images without skin collimation have potential for use in patient electron beam treatment planning. However, the small distortion in images with skin collimation makes them unsuitable for clinical use.

PACS: 87.53.Tf, 87.59.Fm, 87.53.Fs

## I. INTRODUCTION

High energy electrons are a standard radiation therapy for superficial tumors in the head and neck. Those most often treated with electrons and using skin collimation include basal cell and squamous cell carcinomas of the eyelids, lip, tip of nose and ear.^(1)^ Skin (surface) collimation provides optimal sparing of critical structures adjacent to the target by minimizing dose due to scattered electrons. Skin collimation is particularly useful in conjunction with off‐surface bolus (scatter plate) to restore the sharp beam penumbra after the beam has been scattered and the energy degraded by the scatter plate.^(2)^

The size and positioning of skin collimation within the beam are essential for accurate patient treatment.^(3)^ The inner edge of the skin collimation must be sufficiently inside the penumbra cast by the geometric (light) field edge defined by the electron applicator insert to ensure uniform dose to the planning target volume (PTV). Its outer edge must extend well outside the geometric field edge to minimize scatter electron dose outside the treatment area. The thickness of the skin collimation must exceed the minimum thickness needed to stop the electrons, but should not be so thick as to cause discomfort to the patient or produce excessive scatter from the aperture edges.

**Figure 1:**
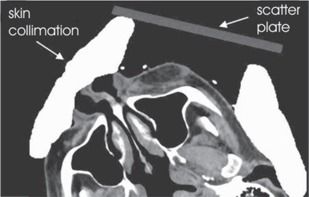
CT image of a patient treated using electrons with skin collimation and scatter plate. The skin collimation and scatter plate were added in the treatment planning system using manual contouring tools.

Treatment planning using skin collimation would benefit from tools that could automatically design skin collimators and off‐surface bolus. However, current commercial treatment planning systems used in our clinic, including the Pinnacle^3^ 7.4f (Philips Medical Systems, N.A., Bothwell, WA) used in this study, lack these features. In light of these deficiencies, the method used in our clinic for treatment planning with skin collimation is to manually draw the collimation onto each of the transverse CT images as an anatomic structure (cf., Fig. 1). A density of Pb (11.3 g/cm^3^) or Cerrobend (9.4 g/cm^3^) is then assigned to the structure. One surface of the skin collimator can be contoured by following the outline of the patient surface, which is clearly seen on the CT image (Fig. 1). The other surface is constructed by ensuring the thickness is sufficient to stop the electrons in the beam. Most importantly, the beam‐defining edges of the collimator must be correct on every slice, matching the patient setup. In general, the process of drawing the skin collimation in the treatment planning system is approximate, tedious, and inefficient.

A better method for incorporating skin collimation into treatment planning that would be more accurate and efficient would be to CT scan the patient with the constructed skin collimation in place so that it appears in the images. However, the high attenuation of kilovoltage photons by high‐Z materials is not adequately modeled by conventional CT image reconstruction algorithms, resulting in extreme distortions and streak artifacts.^(4)^ Dose calculations would therefore be inaccurate if done using kVCT images acquired with skin collimation in place.

**Figure 2:**
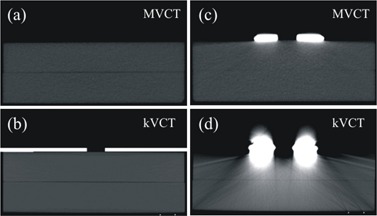
Examples of MVCT and kVCT images of a homogeneous rectilinear phantom scanned without Cerrobend skin collimation a) MVCT and b) kVCT; and with a 3×3 cm skin collimator c) MVCT and d) kVCT. The kVCT image scanned without skin collimation includes a manually‐drawn 3×3 cm skin collimator.

It has been observed that high‐density materials (e.g., aluminum, titanium, and copper) produce dramatically less distortion in MVCT images than in kVCT images.^5^, ^6^ Megavoltage photons are less attenuated by high‐Z materials, so that more photons penetrate the skin collimation and are registered by the CT detectors. This produces more accurate reconstruction results with conventional algorithms, giving more accurate CT numbers and less streaking artifacts. The TomoTherapy Hi‐Art system (TomoTherapy Inc., Madison, WI) has an onboard MVCT scanner (3.5 MV) for image guided radiation therapy (IGRT).^(7)^ Fig. 2 illustrates the reduction in artifacts in imaging of a

Cerrobend collimator on a CIRS Plastic Water (Computerized Imaging Research Systems Inc., Norfolk, VA) phantom with MVCT (Fig. 2c) as compared to kVCT (Fig. 2d). For comparison, all four images in Fig. 2 show the entire phantom at approximately the same magnification and position and are displayed with the same window/level settings.

We hypothesized that MVCT images taken with skin collimation in place are sufficiently accurate for use in electron beam treatment planning. We tested this hypothesis using a simple rectilinear phantom, an anthropomorphic phantom, and simple skin collimation. The utility of dose calculation on the MVCT images was assessed by comparison to doses calculated on kVCT images using the same pencil‐beam dose algorithm.

## II. METHODS

Electron beam dose calculations using a single electron beam were generated using MVCT images and compared to the same calculations using kVCT images. Comparison calculations were performed using a homogeneous rectilinear phantom composed of CIRS Plastic Water and a CIRS model 605 radiosurgery head phantom. Initial tests used images without skin collimation to determine the inherent consistency of electron beam dose calculations on MVCT images. Skin collimation was then introduced to determine the consistency of dose calculations for this treatment situation. All treatment plans were computed using version 7.4f of the Pinnacle^3^ treatment planning system (TPS). Megavoltage CT images were taken on a TomoTherapy Hi‐Art system with a slice spacing of 2.0 – 4.0 mm and a transaxial resolution of 0.75 mm. Kilovoltage CT images were taken on a General Electric Lightspeed RT scanner (General Electric, Milwaukee, WI) with slice spacing of 2.5 mm and transaxial resolution of 0.7 – 0.78 mm, depending on the field‐of‐view. All electron beams were modeled for a Varian linac.

### A. Determination of density, linear collision stopping power ratio, and linear angular scattering power ratio

In order to convert MVCT numbers to densities, calibrations were performed using a CIRS model 062 electron density phantom. This phantom is composed of an outer ‘torso’ and an inner ‘head’ section and has a series of inserts of known physical densities. For each calibration, the ‘torso’ phantom was scanned two consecutive times using a slice thickness of 2.0 mm on the TomoTherapy Hi‐Art system. The resulting images were transferred to the Pinnacle^3^ TPS. A circular region‐of‐interest (ROI) was created within the image of each insert. The ROI was at least 5 mm in diameter and no bigger than 15 mm in diameter in order to sample a large number of pixels at the center of an insert and to avoid CT number variations at its edges. For each ROI, the mean CT numbers from the two scans were averaged. The resulting data were used to create a CT number‐to‐density correlation table. To remove possible effects of daily fluctuations in CT numbers on experimental results, a MVCT calibration was performed on each of the four experiment days.

Electron beam doses were calculated on Pinnacle^3^, which uses a pencil‐beam dose algorithm that is based on the 3D implementation by Starkschall et al. ^(8)^ of the Hogstrom algorithm.^(9)^ The calculation uses ratios of linear collision stopping power and linear angular scattering power relative to those of water for each CT voxel. In Pinnacle^3^, the stopping and scattering power ratios are determined for each voxel from a look‐up table that correlates them to the physical density of that voxel (Table 1). Because Pinnacle^3^ version 7.4f limits density values in this table to a maximum of 3 g/cm^3^, densities greater than this value may be entered into the table, but are treated as if they were 3 g/cm^3^. Clearly, this is not large enough to correctly simulate Cerrobend. Therefore, a density of 2.9 g/cm^3^ was entered into the table along with stopping and scattering power ratios suitable for Cerrobend. Table 1 is based on the one for human tissues originally developed by Hogstrom et al.^(9)^ except for the last entry.

**Table 1:** Lookup table used by Pinnacle^3^ to determined electron beam transport parameters from physical density.

*Physical density (g/cm^3^)*	*Collision stopping power ratio*	*Angular scattering power ratio*
0.000	0.001	0.001
0.291	0.311	0.292
0.927	0.933	0.729
1.000	1.027	0.912
1.047	1.051	1.040
1.100	1.098	1.135
1.427	1.422	1.863
1.940	1.940	3.026
2.900	11.300	11.900

Although phantom materials may accurately duplicate the densities of water and tissues, they do not usually replicate the chemical composition, and small chemical changes can have significant effects on electron transport and even on the attenuation of low‐energy photons. CIRS Plastic Water was used for the rectilinear homogeneous phantom experiments. Its density, reported by the manufacturer to be 1.03 g/cm^3^, was confirmed by independent measurement. Assuming a composition equivalent to polystyrene, its mass stopping power was estimated to be $1.971\;\text{MeV}\;g^{-1}\;{\text{cm}}^{2}$ for 10 MeV electrons, which gave a linear stopping power relative to that for water of 1.002. This linear stopping power ratio corresponds to a physical density of 0.981 g/cm^3^ in the Pinnacle^3^ conversion table (Table 1). The measured MVCT number for CIRS Plastic Water was 1032, which correlates to a density of 1.02 g/cm^3^ based on the calibration with the electron density phantom. Therefore, in the CT number‐to‐density conversion table, densities for tissues from adipose to liver were scaled by a factor of 0.964 (0.981/1.02). This change produced the correct correlation between CT number and stopping and scattering power ratios. Similar corrections were made for the CIRS anthropomorphic radiosurgery phantom and for the kVCT conversion tables.

In the present study it was not our objective to evaluate the degree of accuracy of the pencil beam algorithm in Pinnacle^3^. Rather, it was to assess whether the use of MVCT scan data with skin collimation on the phantom (proposed future clinical application) produced the same dose distribution as did the kVCT scan data with the skin collimation added as a structure (current clinical practice). Nonetheless, to perform this evaluation, it was necessary to ensure that under normal circumstances (with no skin collimation) that the dose distributions calculated using the MVCT and kVCT data for the Plastic Water, agreed.

### B. Rectilinear homogeneous phantom, no skin collimation

Two slabs of 30×30 times 5 cm CIRS Plastic Water were stacked to produce a flat surface, water‐like phantom. MVCT images were taken at a slice thickness of 4.0 mm. Kilovoltage CT images were taken at 2.5 mm slice thickness. Central transverse slices through the images sets are shown in Figs. 2a and 2b, respectively. For each image set, doses were calculated for 9 MeV and 16 MeV electron beams normal to the surface at a source‐surface distance (SSD) of 100 cm. The 10×10 cm applicator was used with no additional blocking, and the prescription dose was 100 cGy at a depth of $D_{\text{max}}$ on the central axis. Doses were compared only in the transverse plane through the central axis of the beam.

Beam SSDs and dose distributions for the two image sets were registered using the posterior edge of the phantom, where any image distortions due to surface collimation would be minimal. First, the couch was removed from each image set by contouring a region‐of‐interest (ROI) aligned to the surface of the couch and setting its density to 0.0 g/cm^3^. Then, a posterior beam was created with an SSD of 100 cm. A point‐of‐interest (POI) was set at the beam isocenter, and its coordinates relative to the image set were determined. Finally, identical dose grids on the two image sets were aligned using the POIs. The Pinnacle^3^ default threshold value of 0.60 g/cm^3^ was used for the posterior surface definition.

Comparisons of calculated doses in the homogeneous phantom for field sizes smaller than 10×10 cm and without skin collimation were unnecessary. Because the two dose distributions had such excellent agreement, not only along central axis (region of lateral side scatter equilibrium), but also in the penumbral region (region of lateral side scatter disequilibrium), there was no reason to expect any less agreement for smaller fields (e.g., 3×3 cm) where the central‐axis region can have disequilibrium, as well as the penumbra.

### C. Anthropomorphic head phantom, no skin collimation

A dose comparison was done using the CIRS model 605 radiosurgery head phantom. MVCT and kVCT images were taken with a slice thickness of 2.5 mm. The standard settings for MVCT imaging were 2.0, 4.0, and 6.0 mm. However, it is possible to change system parameters in order to obtain other slice thicknesses, and this was done in order to have the MVCT slice thickness match the kVCT slice thickness. Because of surface contour changes in the superior‐inferior (SI) direction, we did not want dose comparisons to be complicated by different volume averaging of image pixels with different SI dimensions.

Two simple single‐beam treatment plans were created, one using 9 MeV electrons and the other using 16 MeV electrons. The beams were unblocked with a 10×10 cm field size at 100 cm SSD and a prescription of 100 cGy given dose (central axis dose maximum in water for the same conditions) delivered at a gantry angle of 125°. The location of the central axis and the oblique gantry angle were chosen so that the beams traversed both bone and air heterogeneities in a region that had anatomy conducive to accurate registration of the image sets. The MVCT‐kVCT registration was done manually using the tools available in Pinnacle^3^. Doses were compared in the transverse plane containing the isocenter of the beam.

### D. Rectilinear homogeneous phantom, skin collimation

The rectilinear homogeneous slab phantom of CIRS Plastic Water was MVCT scanned nine times with different Cerrobend skin collimators on its surface using a slice thickness of 4 mm. The nine skin collimators had field sizes of 3×3, 6×6, and 10×10 cm and thicknesses of 6, 8, and 10 mm, which in clinical practice accommodate beam energies of 6 MeV to 16 MeV. In order to simulate this treatment with kVCT images, the phantom was also scanned on the Lightspeed RT unit without skin collimation, and then collimators were drawn onto the images using the contouring tools in Pinnacle^3^. Figs. 2b and 2c show the transverse central plane of the kVCT image with the 3×3 cm, 6 mm thick skin collimator drawn onto it and the same plane imaged with MVCT with the collimator in place, respectively. Because the kVCT image was taken without skin collimation, it was an accurate representation of the phantom and mimics currently accepted clinical practice.

Twelve treatment plans were generated. Each plan consisted of a single anterior beam delivering a prescribed dose of 100 cGy to a depth of $D_{\text{max}}$ on the central axis of an unblocked field 10×10 cm with the selected energy (100 MU). For each skin collimator, plans were computed for energies of 6, 9, 12, and 16 MeV using Cerrobend thicknesses of 6, 6, 8, and 10 mm, respectively. The 10×10 cm applicator was used for the 3×3 and 6×6 cm skin collimators. The 15×15 cm applicator was used for the 10×10 cm skin collimator.

It was expected that skin collimation would produce some small distortions in the MVCT images. These distortions would be manifest as errors in the CT numbers and would, consequently, produce differences in calculated doses relative to doses calculated using kVCT images. In order to better understand how these distortions might influence dose calculation, density was measured as a function of depth along the central axis of the treatment beams. The effect of the inherent noise in the images was reduced by averaging CT numbers within small ROIs created on the kVCT and MVCT image sets. The ROIs were cuboids with transverse dimensions of 1.0×0.4 cm and SI thickness equal to the CT slice thickness. A total of 20 ROIs, extending to a depth of 8.0 cm were created. The mean density was computed in each ROI and assigned to the depth at the center of the ROI. Mean density as a function of depth was compared for each of the collimated MVCT images to the corresponding values in the kVCT images. The differences represented a measure of the distortion in the CT values of the MVCT images. Electron beam doses along the central axis were plotted as a function of radiological depth to remove the effect of density distortion. Radiological depth as a function of physical depth was computed by integrating the mean density from the surface to the physical depth.

### E. Evaluation of MVCT doses

All analyses were performed and reported using absolute doses. Agreement between each MVCT dose calculation and the corresponding kVCT dose calculation was assessed by comparing the respective dose distributions in the central axis transverse planes. In the presence of skin collimation, because the MVCT scanner resolution in the direction of couch motion differs from that of the kVCT scanner, dose agreement in a sagittal plane should be worse than that reported in this study for the transverse plane. Furthermore, because clinical skin collimators are irregularly shaped, these effects should also adversely affect dose comparisons in transverse planes. Results will show that dose comparisons for rectilinear skin collimation in transverse planes are not clinically acceptable. Therefore, these more advanced dose comparisons were not performed in the current study.

For the dose comparisons in the transverse planes, both with and without the square skin collimation, the 2D dose matrices were exported from Pinnacle^3^ into SigmaPlot and MATLAB (The MathWorks Inc., Natick, MA) for analysis. Agreement between each MVCT dose calculation and the corresponding kVCT dose calculation was assessed by comparing the respective dose distributions in the central axis transverse planes. Isodose curves and central axis depth‐dose curves were superimposed for visual comparison and quantitative analysis. In the uniform dose region, dose differences ($D_{\text{MVCT}}$ ‐ $D_{\text{kVCT}}$) were computed and expressed as percent differences from the kVCT image doses. The uniform dose region was defined as the area with doses ≥ 90% of the prescription dose, the area typically containing the PTV and exclusive of the surface build‐up region and penumbral regions. In the high dose‐gradient regions, distance‐to‐agreement (DTA) was computed and evaluated. The high dose‐gradient regions were defined as the penumbra and distal falloff areas with doses between 10% and 90% of the prescription dose. DTA was defined as the minimum distance from a dose point in the MVCT image to a point in the kVCT image with the same dose.^10^, ^12^ Each point in the MCVT dose matrix was tested against a square region in the kVCT image of size 1.7×1.7 cm centered on the location of the test point. In the kVCT dose test region, the distances were computed from the center to the interpolated locations of the test dose in all the rows and columns. The minimum distance was taken as the DTA.

## III. RESULTS

### A. MVCT number to density conversion

Four MVCT number‐to‐density conversion tables (each an average of two scans) were measured over a period of about 6 months. Each table was used to calculate doses for the images acquired on that day. The measured tables show good consistency over the 6 month period that the data were acquired (Table 2). The largest standard deviation was 8 CT numbers, which represents an uncertainty of less than 0.01 g/cm^3^ in physical density.

**Table 2:** MVCT number‐to‐density data acquired over a 6‐month period for CIRS model 062 electron density phantom.

*Material*	*Physical density (g/cm^3^)*		*CT number*			Mean(±SD)
		*Day 1*	*Day 2*	*Day 3*	*Day 4*	
air	0.001	1	4	4	1	3±1.7
lung (inhale)	0.195	224	232	232	232	230±4.0
lung (exhale)	0.495	496	508	504	507	504±5.4
adipose	0.967	970	974	974	977	974±2.9
breast	0.991	1004	1011	1008	1013	1009±3.9
water	1.000	1028	1020	1017	1019	1021±4.8
muscle	1.062	1052	1064	1061	1065	1061±5.9
liver	1.071	1062	1077	1076	1080	1074±8.0
trabecular bone	1.161	1137	1148	1146	1151	1146±6.0
dense bone	1.609	1500	1508	1506	1507	1505±3.6

### B. Rectilinear homogeneous phantom, no skin collimation

**Figure 3:**
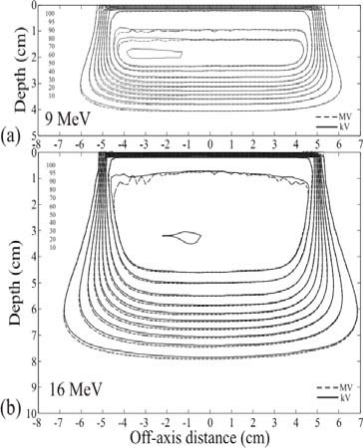
Isodose curves computed with MVCT and kVCT images for a) 9 MeV and b) 16 MeV beams of 10×10 cm size at 100 cm SSD. Isodose values are cGy. The maximum dose differences were 2.3%, and the maximum DTAs were 2.0 mm for both beams.

Superimposed isodose plots computed for the rectilinear homogeneous phantom without skin collimation are shown in Fig. 3 for 10×10 cm fields of energy 9 and 16 MeV. They demonstrated clinically acceptable agreement between MVCT and kVCT‐ based Pinnacle^3^ dose calculations. For the 9 MeV beam the dose differences in the uniform dose region ranged from −2.3% to 1.7%, and for the 16 MeV beam the differences ranged from −2.3% to 1.2%. The largest dose differences were found at the edges of the high dose region. The maximum DTAs in the high dose‐gradient regions were 2.0 mm for the both beams.

### C. Anthropomorphic head phantom, no skin collimation

**Figure 4:**
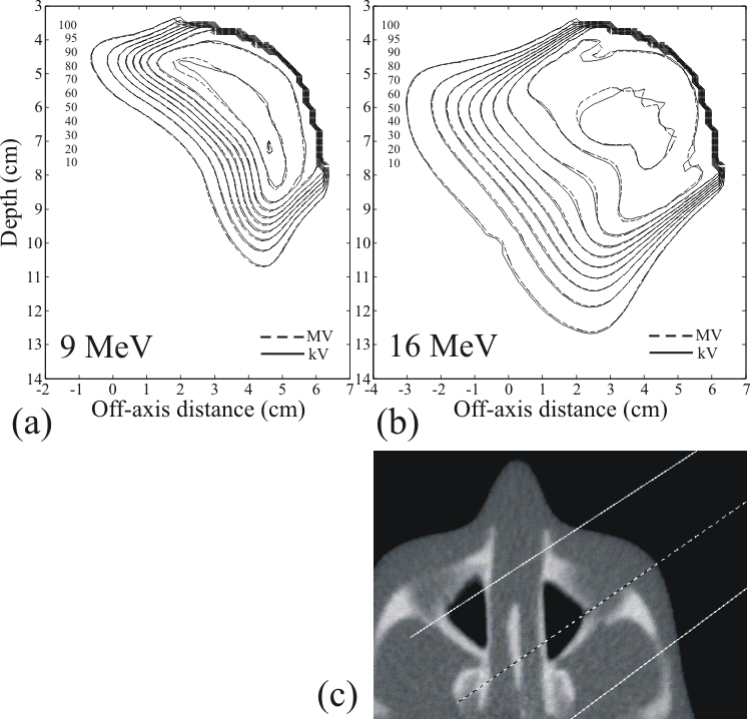
Isodose curves for a) 9 MeV and b) 16 MeV beams computed for the anthropomorphic head phantom. In c) an MVCT image of the phantom through the central axis of the left anterior oblique beam is shown. Isodose values are cGy. The maximum dose differences were 2.8% and 4.5%, respectively. The maximum DTAs were 2.0 mm for both beams.

The superimposed isodose plots computed for the anthropomorphic head phantom, shown in Fig. 4 for the 9 and 16 MeV beams, demonstrated clinically acceptable agreement between MVCT and kVCT‐ based Pinnacle^3^ dose calculations. For the 9 MeV beam the dose differences in the uniform dose region ranged from −1.3% to 2.8%, and for the 16 MeV beam the differences ranged from −4.5% to 2.2%. The maximum DTAs in the high dose‐gradient regions were 2.0 mm for both beams.

### D. Rectilinear homogeneous phantom, skin collimation

**Figure 5:**
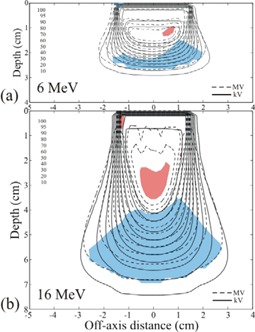
Isodose plots of the dose distributions from a) 6 MeV and b) 16 MeV electrons in the rectilinear homogeneous phantom calculated using MVCT images with 3×3 cm skin collimation, compared to doses calculated using kVCT images with drawn collimation. Isodose values are cGy. The red areas are the regions of uniform dose that have absolute dose differences greater than 5%, and the blue areas are the regions of high dose‐gradient that have DTA values greater than 2 mm.

**Figure 6:**
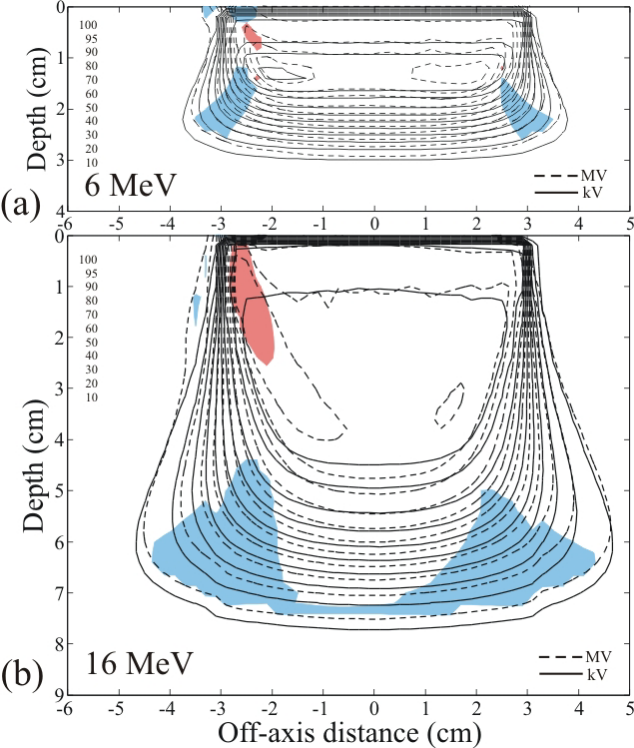
Isodose plots of the dose distributions from a) 6 MeV and b) 16 MeV electrons in the rectilinear homogeneous phantom calculated using MVCT images with 6×6 cm skin collimation, compared to doses calculated using kVCT images with drawn collimation. Isodose values are cGy. The red areas are the regions of uniform dose that have absolute dose differences greater than 5%, and the blue areas are the regions of high dose‐gradient that have DTA values greater than 2 mm.

**Figure 7:**
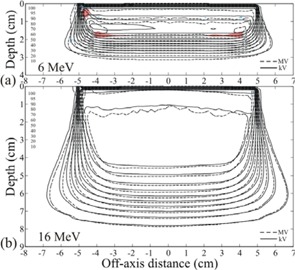
Isodose plots of the dose distributions from a) 6 MeV and b) 16 MeV electrons in the rectilinear homogeneous phantom calculated using MVCT images with 10×10 cm skin collimation, compared to doses calculated using kVCT images with drawn collimation. Isodose values are cGy. For the 6 MeV electrons, the red area is the region of uniform dose that has absolute dose differences greater than 5%, and the blue area is the region of high dose‐gradient that has DTA values greater than 2 mm. For the 16 MeV electrons the regions of dose difference greater than 5% and DTA greater than 2 mm are not discernable.

In Figs. 5–7, the isodose plots of the dose distributions computed for the MVCT images of the rectilinear homogeneous phantom with skin collimation are compared to the isodose plots of the dose distributions computed for the kVCT images with drawn skin collimation. The isodose plots are taken through the central axis transverse planes of the 6 MeV and 16 MeV beams, and Figs. 5–7 correspond to skin collimation of 3×3, 6×6, and 10×10 cm, respectively. The maximum dose differences in the uniform high‐dose region and the maximum DTAs in the high dose‐gradient regions for the central axis planes for all energies (6, 9, 12, and 16 MeV) and field sizes (3×3, 6×6, and 10×10 cm) studied are shown in Table 3. For the MVCT dose calculations, only the higher energy beams (12 and 16 MeV) with large field size (10×10 cm) were close to matching the kVCT dose calculations to within 5% and 2 mm DTA, e.g. 5.2% and 2.1 mm DTA at 16 MeV. The worst case overall was the 3×3 cm field at 12 MeV, where the MVCT calculation differed from the kVCT calculation by 12.2% and 7.3 mm DTA.

**Table 3:** Maximum dose errors in the uniform‐dose regions and maximum DTAs in high dose‐gradient regions for electron beam calculations in the MVCT images of the homogeneous phantom with skin collimation.

*Energy*	*Maximum dose error (%) / Maximum DTA (mm)*		
	$3\times 3\;{\text{cm}}^{2}$	$6\times 6\;{\text{cm}}^{2}$	$10\times 10\;{\text{cm}}^{2}$
6 MeV	−6.4%/3.7 mm	−6.4%/2.4 mm	9.1%/2.3 mm
9 MeV	6.8%/3.7 mm	−5.3%/3.0 mm	7.1%/2.6 mm
12 MeV	−12.2%/7.3 mm	−5.3%/3.4 mm	−4.7%/2.7 mm
16 MeV	−7.5%/6.7 mm	13.2%/3.2 mm	−5.2%/2.1 mm

**Figure 8:**
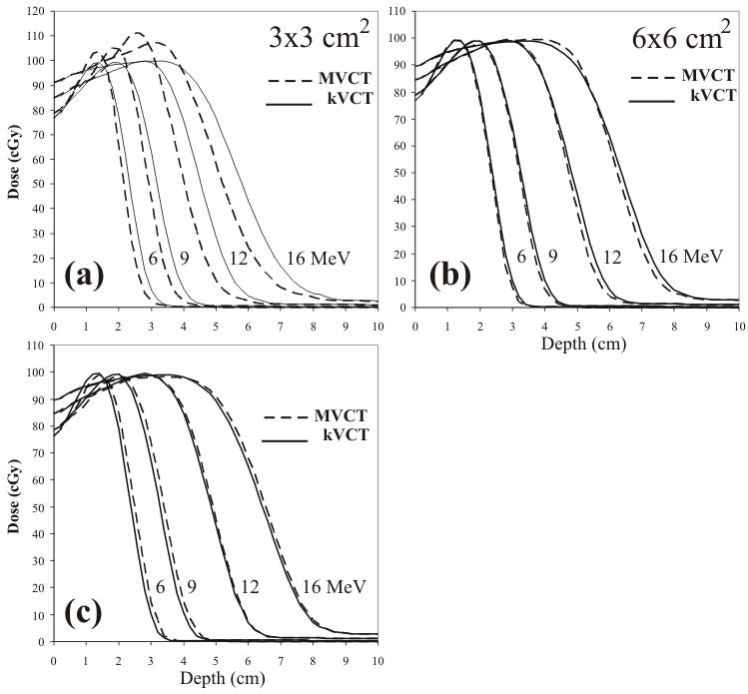
Comparison of central axis depth doses for dose distributions calculated using MVCT image (dashed lines) and kVCT images (solid lines) for all beam energies (6, 9, 12, and 16 MeV) and all field sizes defined by skin collimation; a) 3×3 cm, b) 6×6 cm, and c) 10×10 cm. Cerrobend collimator thicknesses were 6 mm (6 and 9 MeV), 8 mm (12 MeV), and 10 mm (16 MeV). All beams were delivered with 100 MU (≈ 100 cGy given dose).

Central axis depth‐dose comparisons of MVCT with kVCT‐calculated doses for the four beam energies (6, 9, 12, and 16 MeV) and three skin collimators (3×3, 6×6, and 10×10 cm) are shown in Fig. 8. In general, in the dose falloff region (90%–10%) the central axis doses for the MVCT images significantly underestimate that for the kVCT images for the 3×3 cm field size, slightly underestimate it for the 6×6 cm field size, and slightly overestimate it for the 10×10 cm field size, as defined by the skin collimation. The central axis depth doses show remarkably good agreement for the 6×6 cm field at low energies (6 MeV and 9 MeV) and for the 10×10 cm field at high energies (12 MeV and 16 MeV). Also, central axis depth‐dose comparisons of the MVCT and kVCT calculations show that the MVCT central axis dose maximum overestimates the kVCT central axis dose maximum and underestimates the dose in the falloff region. This is particularly significant and clinically unacceptable for the 3×3 cm field.

**Figure 9:**
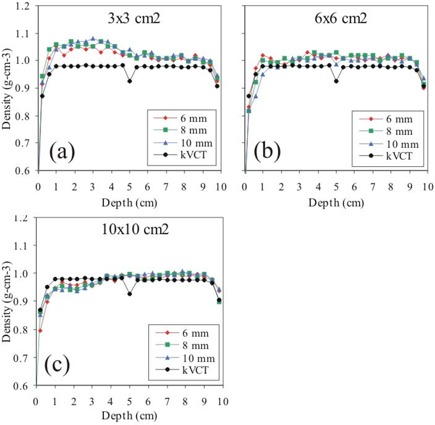
Phantom density as a function of depth along the beam central axis for the MVCT images with each of the different skin collimator thicknesses (6, 8, and 10 mm) compared to density of the reference kVCT image for a) 3×3 cm, b) 6×6 cm, and c) 10×10 cm field sizes.

To better understand the observed depth‐dose differences, we studied MVCT number versus depth. In Fig. 9, the mean phantom density along the central axis in the MVCT images is shown as a function of depth for the different field sizes (defined by the skin collimator) and different skin collimator thicknesses. For comparison, the mean density along the central axis as a function of depth is shown for the kVCT image without skin collimation. The density along the central axis versus depth for the MVCT image without skin collimation is not shown. The graph of MVCT densities on the central axis with no skin collimation lies virtually on top of the corresponding graph of the kVCT densities. This is evident from the excellent agreement on the central axis of MVCT doses to kVCT doses without the presence of skin collimation (Fig. 3). For the 16 MeV beam, the distal 10% dose falls at an interpolated depth of 7.85 cm on the kVCT image and at an interpolated depth of 7.91 on the MVCT image. This corresponds to a difference of 0.06 cm in integrated depth over a distance of 7.85 cm; a difference of 0.8% in the density of each pixel in the path, corresponding to a change in CT number of 8 units out of 1000.

The largest effects are seen in the first 4 cm of depth. For the 3×3 cm field size, MVCT densities in this region are much greater than those in the kVCT images, increase with skin collimator thickness, and reach a maximum increase of about 10% for the 10 mm thickness. For the 6×6 cm field size, MVCT densities change significantly less with depth or collimator thickness and are only slightly greater than those in the kVCT images. For the 10×10 cm field size, the mean MVCT densities are up to 3% lower than the kVCT densities in the first 4 cm of depth and vary little with collimator thickness. The skin collimation produces greater distortions at shallower depths, not an unexpected result.

**Figure 10:**
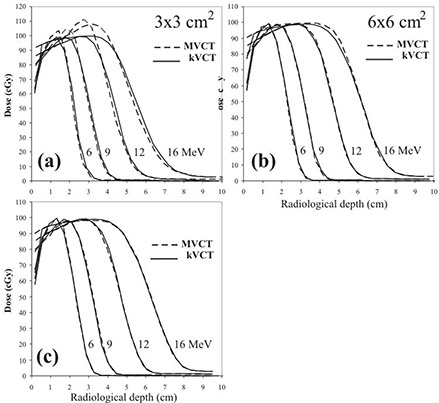
Dose versus radiological depth along the beam central axis for all energies (6, 9, 12, and 16 MeV) and skin collimation of a) 3×3 cm, b) 6×6 cm, and c) 10×10 cm.

To more directly examine the effect of distortion in phantom density values on calculated dose, dose versus radiological depth along the central axis is plotted in Fig. 10 for each beam energy (6, 9, 12, and 16 MeV) and skin collimator (3×3, 6×6, and 10×10 cm) combination. The reference kVCT depth‐dose curves are also computed using radiological depth in this figure. Almost all of the previously observed dose differences in the central axis depth‐dose falloff region for the intermediate and large field sizes are eliminated. For the small field size, the small error (<2mm) in the distal falloff region is reduced, but not eliminated, and differences in the peak remain.

## IV. DISCUSSION

The results indicate that MVCT scans show promise for patient electron beam dose planning without skin collimation. For beam energies of 9 MeV to 16 MeV and field sizes up to 10×10 cm, doses in the homogeneous phantom were within 3% in the uniform dose region that would cover the target and within 2 mm DTA in the gradient regions that might abut critical structures. In the anthropomorphic head phantom, the dose differences were small for the 9 MeV beam (less than 3%), but slightly larger for the 16 MeV beam (up to 4.5%). For both energies, the maximum DTA was 2 mm. However, for a patient or anthropomorphic phantom, it is not certain that kVCT images are a more faithful representation than MVCT images. Even bones can produce minor streak artifacts in kVCT images. Therefore, without dosimetric measurements, the true accuracy of doses in heterogeneous MVCT images cannot be determined. However, the use of MVCT images with skin collimation for electron beam dose planning is clearly not supported by our data. There were significant differences between doses calculated on MVCT images with skin collimation and doses calculated on kVCT images with skin collimation digitally inserted.

**Figure 11:**
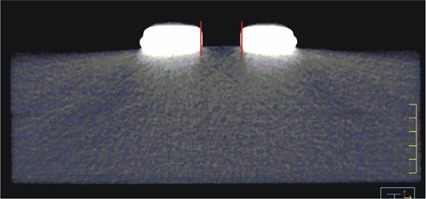
Axial MVCT image showing distorted (rounded) edges of Cerrobend skin collimation (3×3cm, 10mm). The vertical red line segments represent the true locations of the collimator edges.

Although the MVCT images of the phantom with skin collimation were much better than comparable kVCT images (Fig. 1), there remained some distortion in the images. This distortion was not apparent as streaking, but as errors in CT values, distortions in the skin surface, and distortion in the shape of the surface of the skin collimator. Fig. 11, a close‐up of the phantom with the 3×3 cm field size collimator on the surface, illustrates these phenomena. The distortion in the shape of the inner edge of the skin collimator is evident by comparing its edge with the red lines that delineate the actual straight edges of the collimator. The impact of the distortion in the shape of the interior edges of the skin collimator is that electron pencil beams at the edge can penetrate it and scatter incorrectly. The apparently rounded edges cause the pencil beam algorithm to increase the scatter from the inner skin collimator surfaces toward the center of the beam, which increases the superficial dose at the expense of dose at depth. The result is the peaking seen at shallow depths and the reduced penetration for the small beam (cf. Fig. 10a). The effect is less evident for the larger field sizes, but is still seen as “hot” spots inside the field edges at shallow depths. As seen in Figs. 6 and 7, for the larger skin collimator fields (6×6 and 10×10 cm), the collimator edges are far enough from the central axis that those doses are not affected; however, there is a lobe of increased scatter dose caused by the false edges.

Artifacts in kVCT images from high‐density objects were shown by Williamson et al.^(13)^ to be due primarily to mismatches between the simplified model of CT detector response embodied in the filtered backprojection (FBP) reconstruction algorithm and the physical processes of signal acquisition. They demonstrated that their alternating minimization (AM) iterative algorithm performed significantly better than FBP or other iterative algorithms. However, they found that significant streaking artifacts remained even when model mismatch was completely eliminated. Given the inherent improvement of MVCT imaging over kVCT imaging in the presence of high‐density objects, it seems possible that MVCT imaging combined with AM reconstruction could produce distortion‐free images useful for electron beam planning with skin collimation.

## V. CONCLUSIONS

It was found that using Hi‐Art MVCT images of a homogeneous phantom, doses calculated for electron beams of 9 MeV to 16 MeV and field sizes up to 10×10 cm were consistent with doses calculated using kVCT images to within 3% in the uniform dose region that would cover the target and to within 2 mm DTA in the gradient regions that might abut critical structures. For a heterogeneous head phantom, electron beam calculations with MVCT images were consistent with kVCT image calculations to within 3% for a 9 MeV beam and within 5% for a 16 MeV beam, both with maximum DTA of 2 mm. These results suggest that MVCT images without skin collimation have potential for use in patient electron beam treatment planning.

When MVCT images were taken with skin collimation on the phantom, residual distortions in the phantom density and distortions in the images of the skin collimation edges produced differences that were greater than ± 5% and DTAs that were greater than ± 2 mm compared to kVCT images with skin collimation digitally inserted. Although Hi‐Art MVCT images taken with skin collimation in place have dramatically less distortion than comparable kVCT images, the small residual distortion makes them unsuitable for clinical use.

## ACKNOWLEDGMENTS

This work was supported in part by a research agreement with TomoTherapy Inc. and by the Biological Computation and Visualization Center, LA Board of Regents Health Excellence Fund grant # HEF (2000–2005)‐01. Neither funding source was involved in designing the study, analyzing the data, or preparing the manuscript.
